# Exploring spatiotemporal patterns of COVID-19 infection in Nagasaki Prefecture in Japan using prospective space-time scan statistics from April 2020 to April 2022

**DOI:** 10.1186/s13690-022-00921-3

**Published:** 2022-07-26

**Authors:** Yixiao Lu, Guoxi Cai, Zhijian Hu, Fei He, Yixian Jiang, Kiyoshi Aoyagi

**Affiliations:** 1 Department of Public Health, Nagasaki University Graduate School of Biomedical Sciences, Nagasaki, 852-8523 Japan; 2 Public Health and Hygiene Research Department, Nagasaki Prefectural Institute of Environment and Public Health, Nagasaki, 856-0026 Japan; 3 Department of International Health and Medical Anthropology, Institute of Tropical Medicine (NEKKEN), Nagasaki University, Nagasaki, 852-8523 Japan; 4 Department of Epidemiology and Health Statistics, Fujian Provincial Key Laboratory of Environment Factors and Cancer, School of Public Health, Fujian Medical University, Fuzhou, 350122 Fujian Province China

**Keywords:** COVID-19, Emerging clusters, Disease surveillance, Space-time pattern, SaTScan

## Abstract

**Background:**

Up to April 2022, there were six waves of infection of coronavirus disease 2019 (COVID-19) in Japan. As the outbreaks continue to grow, it is critical to detect COVID-19’s clusters to allocate health resources and improve decision-making substantially. This study aimed to identify active clusters of COVID-19 in Nagasaki Prefecture and form the spatiotemporal pattern of high-risk areas in different infection periods.

**Methods:**

We used the prospective space-time scan statistic to detect emerging COVID-19 clusters and examine the relative risk in five consecutive periods from April 1, 2020 to April 7, 2022, in Nagasaki Prefecture.

**Results:**

The densely inhabited districts (DIDs) in Nagasaki City have remained the most affected areas since December 2020. Most of the confirmed cases in the early period of each wave had a history of travelling to other prefectures. Community-level transmissions are suggested by the quick expansion of spatial clusters from urban areas to rural areas and remote islands. Moreover, outbreaks in welfare facilities and schools may lead to an emerging cluster in Nagasaki Prefecture’s rural areas.

**Conclusions:**

This study gives an overall analysis of the transmission dynamics of the COVID-19 pandemic in Nagasaki Prefecture, based on the number of machi-level daily cases. Furthermore, the findings in different waves can serve as references for subsequent pandemic prevention and control. This method helps the health authorities track and investigate outbreaks of COVID-19 that are specific to these environments, especially in rural areas where healthcare resources are scarce.

**Supplementary Information:**

The online version contains supplementary material available at 10.1186/s13690-022-00921-3.

## Background

On January 9, 2020, a novel coronavirus was linked to the outbreak of pneumonia cases in Wuhan, China, and it rapidly spread worldwide within months. The World Health Organization (WHO) officially named this new disease “coronavirus disease 2019” (COVID-19) [1]. By June 12, 2022, over 533 million confirmed cases and over 6.3 million COVID-19 related deaths were reported globally [2]. By early April 2022, Japan had faced the sixth wave of the COVID-19 epidemic [3].

**Fig. 1:**
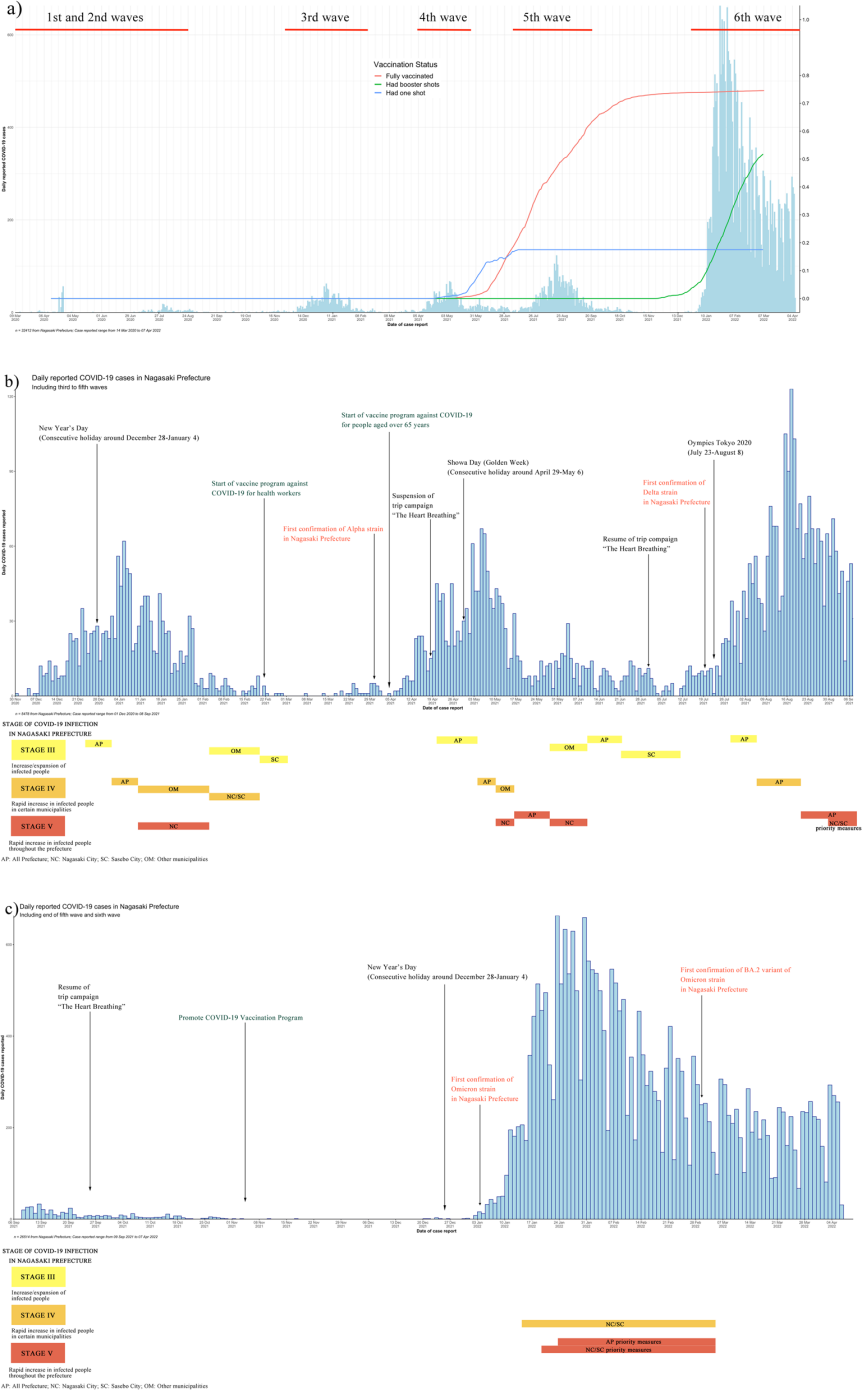
Daily number and epidemiological curve of COVID-19 cases in Nagasaki Prefecture from 2020/4/1 to 2022/4/7. **a**) Daily reported COVID-19 cases in Nagasaki Prefecture from 14 March 2020 to 7 April 2022, **b**) COVID-19 infection stage in the third to fifth waves in Nagasaki Prefecture, and **c**) COVID-19 infection stage in the sixth wave

In Nagasaki Prefecture, the first case of COVID-19 was identified on March 14, 2020. Before December 2020, Nagasaki Prefecture was attacked by several small COVID-19 outbreaks with fewer than 25 cases per day (first wave). Even so, a nationwide state of emergency was declared between April 4, 2020 and May 14, 2020 [4]. In the following four waves of the COVID-19 epidemic between December 2020 and September 2021, the number of daily COVID-19 infections largely increased. During the fifth wave (July 6 to September 27, 2021), the number of daily infections increased by 2.5–3 times compared to the same period the previous year (Fig. 1a). From the beginning of 2022, a new COVID-19 variant, Omicron [5], caused the largest-ever surge, pushing Japan’s daily COVID-19 cases to a new record. In the sixth wave (January 10 to June 10, 2022) of COVID-19 in Nagasaki Prefecture, the number of new COVID-19 confirmed cases had reached its highest in the week (1/24/2022–1/30/2022) (Fig. 1a).

In addition to traditional non-pharmaceutical interventions (NPIs) such as social distancing, travel restrictions, school closures, and so on, the COVID-19 vaccination campaign, and the regional state of emergency were used as two strong measures to control the deteriorating COVID-19 situation.

The COVID-19 vaccine program in Japan started in April 2021 with the immunization of health workers and the older people (> 65 years). Although there was a delay in the vaccine roll-out in Japan comparing to other industrialized countries [6], the Japanese Health Ministry managed to reach a high vaccination rate by September 2021 [7]. In Nagasaki Prefecture, around 75% of the population was fully vaccinated (received two shots in a two-dose vaccine series) (Fig. 1a). A COVID-19 vaccine booster shots (third dose) was initiated in March 2022 [8].

In late June of 2020, the Nagasaki government formulated a certain standard to respond to different stages of COVID-19 infection [9] to ensure a functioning medical care provision system in Nagasaki Prefecture. The detailed indicators of stage judgment and measures to be taken for each stage are in Supplementary material 1. To control the spread of COVID-19, basic countermeasures were implemented in Japan over three phases: domestic spread prevention, preventing the spread of infection, and preventing severe spread. Local governments’ responses are significant in the general prevention of COVID-19 since the situation is unique in different locations [10, 11]. Several studies of mobility change during the first national state of emergency showed Japan had successfully reduced the population density in urban areas without forcing lockdowns [12–14]. There was a significant infection decrease in the third to fifth waves in Nagasaki Prefecture, about 2–3 weeks after a state of emergency was declared (Fig. 1b). Therefore, a better understanding of the transmission dynamics of the pandemic is critical for identifying at-risk groups and locations and supporting the government in making science-based decisions.

Space-time scan statistics is a technique for disease surveillance and early detection that identifies geographic and temporal clusters of elevated disease risk while quantifying cluster strength and statistical significance [15, 16]. The prospective space-time scan statistic is beneficial because it detects active or emerging clusters at the end of the study period and the relative risk for each affected site during the epidemic [17]. Several studies used this method to monitor the COVID-19 epidemic at a large-scale level [18–20]. In a region like Nagasaki Prefecture, with an aging population and diverse geographical conditions, it is of significant value to examine the high-risk areas of transmission in the consecutive waves of COVID-19 to understand the transmission dynamics of high-risk locations and promptly implement control measures that target the vulnerable locations and groups.

This study aimed to identify active clusters of COVID-19 infections in Nagasaki Prefecture and describe the spatiotemporal patterns of high-risk areas in five consecutive different infection periods.

## Methods

### Study area and data

**Fig. 2:**
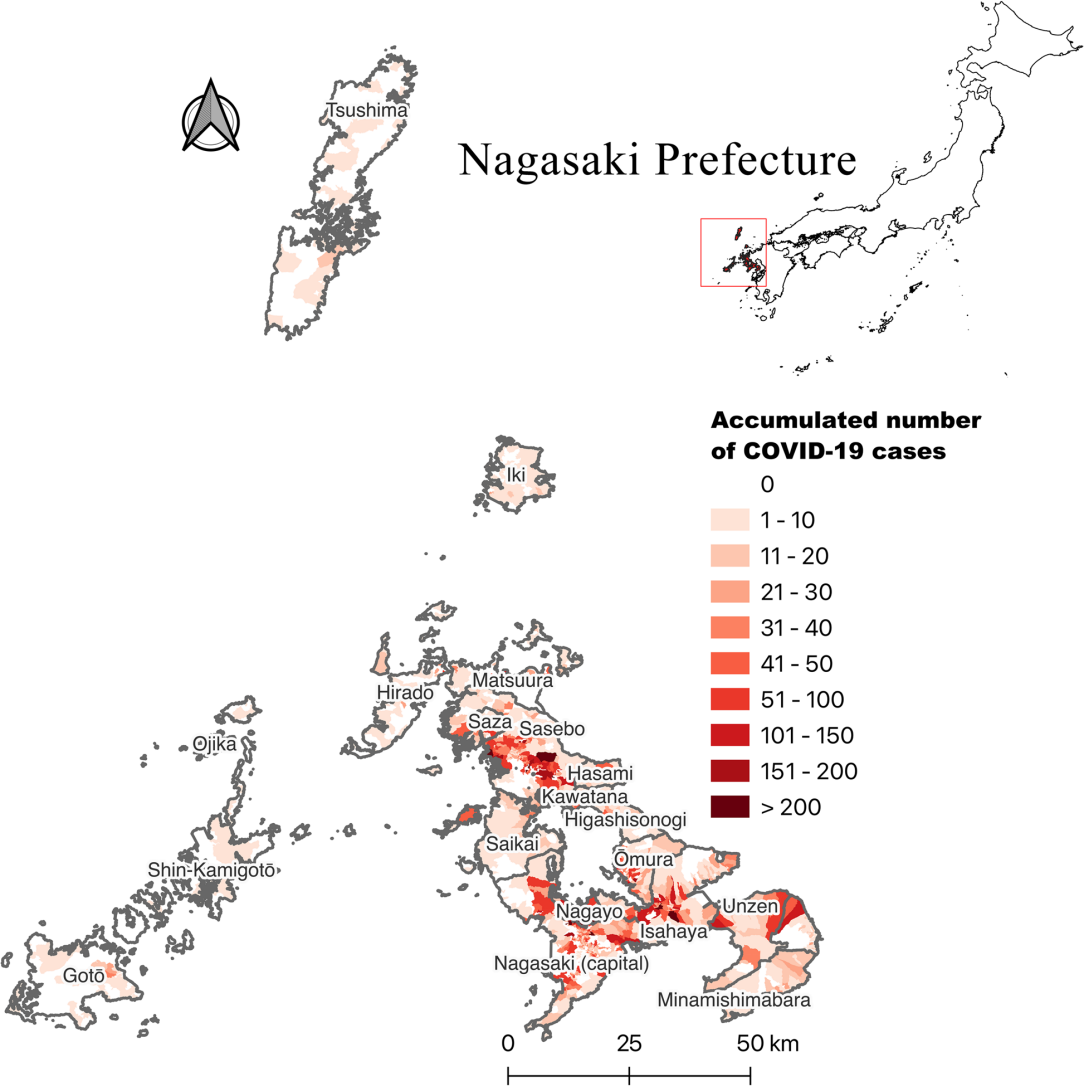
Cumulative number of COVID-19 cases by city/district in Nagasaki Prefecture from 2020/4/1 to 2022/4/7

Nagasaki Prefecture is located on the island of western Kyushu, with a geographic area of 4130 km^2^. It consists of four peninsulas centered around Omura Bay (Kitamatsuura, Nishisonogi, Nagasaki, Shimabara) and three remote islands (Tsushima, Iki, Goto) (Fig. 2).

The information center for infectious diseases of the Nagasaki Prefectural Institute of Environment and Public Health provided the data of daily confirmed COVID-19 cases used in this study. A total of 32,412 confirmed cases had been reported between April 1, 2020 and April 7, 2022. The record of each confirmed case includes age, gender, nationality, type (confirmed case, asymptomatic pathogen holder, suspected case, dead), vaccination status, and residential address. The overall COVID-19 vaccination data in Nagasaki Prefecture were extracted from the GOVERNMENT CIO’s PORTAL, JAPAN (https://cio.go.jp/en/index.php). The machi-level Nagasaki Prefecture population data from 2015 and shapefiles maps of Nagasaki Prefecture/Densely Inhabited Districts (DIDs) [21] used in this study were downloaded from e-Stat (a portal site for Japanese Government Statistics) (https://www.e-stat.go.jp/en).

The residential address data were first de-identified by restricting to a machi-level address, then cleaned by filtering out the records without a valid address and those located outside of Nagasaki Prefecture. In total 29,853 COVID-19 cases with complete address were included in the spatial-temporal analysis. The de-identified residential address was translated into coordinate information (latitude and longitude). We combined the cleaned and translated COVID-19 dataset with the Nagasaki prefectural census data at the machi-level through the “KEY_CODE,” a link code of figures and aggregated data. The entire study period is divided into five analysis periods that consider the incubation time for the disease that ranges from 1 to 14 days [22] and the official determination of each COVID-19 wave (https://www.pref.nagasaki.jp). The five analysis periods were as follows: 1) April 1, 2020 to November 30, 2020, which included the first and second waves; 2) April 1, 2020 to March 31, 2021, which included the third wave; 3) April 1, 2020 to June 30, 2021, which included the fourth wave; 4) April 1, 2020 to September 8, 2021, which included the fifth wave; and 5) April 1, 2020 to April 7, 2022, which included the sixth wave.

### Prospective space-time scan statistics

We used the prospective space-time scan statistic [16] implemented in SaTScan™ (www.satscan.org/) to detect active clusters of COVID-19 cases until the end of each study period. Using this method, the cluster with the maximum likelihood (the most likely cluster) is obtained from several cylindrical candidate clusters with significant likelihood ratios (*p* < 0.05). We assumed the COVID-19 cases follow a Poisson distribution according to the at-risk population, and used the discrete Poisson-based probability model in this study. The model also considers that the population size is static in an area through a certain time. The relative risk (RR) of COVID-19 for each machi and each detected cluster was calculated, to evaluate the risk of COVID-19 in the clustered areas [23].

To prevent the overlapping of candidate clusters and to cover the largest possible high-incidence geographic areas [24], the data from April 1, 2020 to March 31, 2021 were used as the basis for testing. By increasing the spatial scanning windows size and temporal cluster size from 1 to 50%, the spatial and temporal scanning windows were restricted to include 5% or less of the at-risk population and 50% or less of the study period, respectively. Furthermore, each candidate had to include at least two cases and have a minimum of 2 days. The gender and vaccination status (whether or not fully vaccinated) were used for the covariate adjustment.

The above analysis was repeated for five study periods; we then used QGIS 3.20.2-Odense software to generate choropleth maps representing the detected emerging clusters at the machi-level.

## Results

### Basic characteristics of reported COVID-19 cases

The basic characteristics of COVID-19 positive cases are shown in Table 1. In total, 32,412 COVID-19 positive cases from Nagasaki Prefecture were reported by April 7, 2022. Of those, 16,102 (49.7%) were male cases. The median age (IQR) of reported cases was 32 (15–49) years, and the majority of cases (28,164, 86.8%) were under 65 years old. Of all the confirmed cases, 38.3% had been fully vaccinated against COVID-19.

**Table 1:** Characteristics of 32,412 reported COVID-19 cases in Nagasaki Prefecture from 2020/4/1 to 2022/4/7

Characteristics	Overall *n* = 5766	Overall %
**Gender** (370 missing)		
Male	16,102	49.7%
Female	15,940	49.2%
**Nationality**		
Japan	32,240	99.5%
Others	172	0.5%
**Age** (339 missing)	**Median (IQR)**	32 (15–49)
**Age Group** (339 missing)		
0–17	9312	28.7%
18–29	5581	17.2%
30–39	4801	14.8%
40–49	4424	13.6%
50–64	4046	12.5%
65–74	1962	6.0%
75–84	1034	3.2%
≥ 85	913	2.8%
**Diagnostic Results**		
Confirmed cases	28,944	89.3%
APH^a^	3281	10.1%
Suspected cases	54	0.2%
Dead	5	0.02%
Dead (suspected cases)	2	0.002%
**Vaccination Status**		
Not vaccinated	12,422	38.3%
Only one shot	7406	22.8%
Fully vaccinated^b^	12,584	38.8%

*APH*^a^ Asymptomatic pathogen holder, *Fully vaccinated*^b^ Receive the second dose in a two dose COVID-19 vaccine series

### Space-time clusters at machi-level in Nagasaki Prefecture and DIDs: Period 1 (4/1/2020–11/30/2020)

**Fig. 3:**
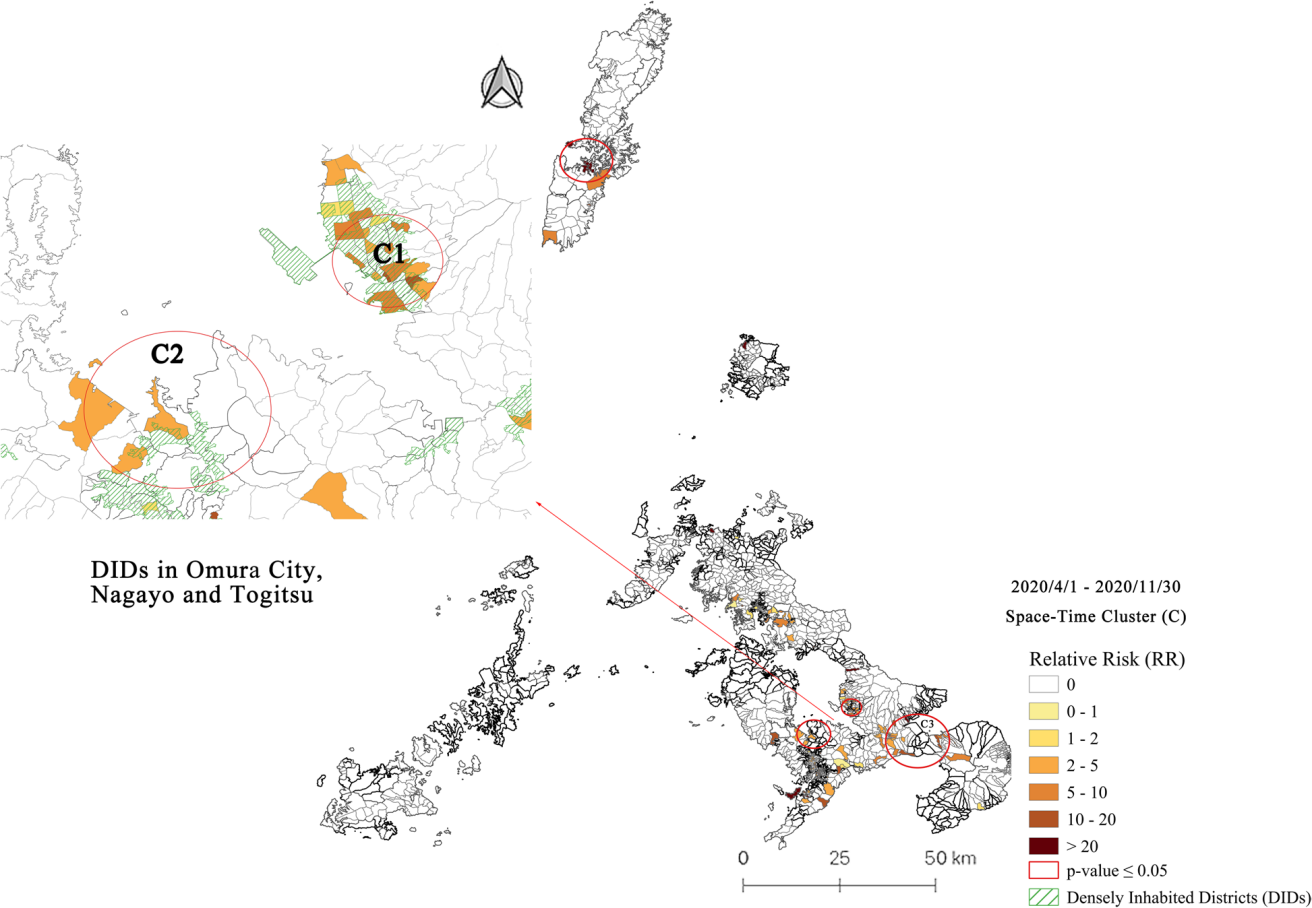
Emerging space-time clusters of COVID-19 cases at machi-level in Nagasaki Prefecture from 2020/4/1 to 2020/11/30

The first study period started on April 1, 2020 and ended on November 30, 2020. In this period, Japan endured two waves of COVID-19, and a nationwide state of emergency was declared between April 16 and May 14, 2020. The first COVID-19 case in Nagasaki Prefecture was reported on Iki Island on March 14, 2020. The most challenging situation in Period 1 occurred on the foreign cruise that anchored near the quay of Nagasaki harbor in April [25]. The prospective space-time scan statistic identified four clusters with statistical significance (*p* < 0.05), shown in Fig. 3 and summarized in Table 2. Clusters 1–3 were located on Nagasaki Prefecture’s mainland (Omura City, Nishisonogi District, and Isahaya/Uzen City), and Cluster 4 on the remote island of Tsushima.

**Table 2:** Characteristics of emerging space-time clusters of COVID-19 cases at machi-level in Nagasaki Prefecture from 2020/4/1 to 2020/11/30

Cluster	Period	Duration (Days)	Radius (km)	Observed Cases	Expected Cases	RR	*P*-value	Machi (Total)	Machi (RR > 1)	Population
1	2020/8/2–2020/11/30	120	2.38	32	5.34	6.61	< 0.001	35	18	50,720
2	2020/8/2–2020/11/30	120	4.03	27	6.99	4.15	< 0.001	17	8	66,404
3	2020/8/2–2020/11/30	120	7.64	23	5.88	4.16	< 0.05	69	16	55,806
4	2020/8/24–2020/11/30	98	6.18	7	0.47	15.37	< 0.05	16	3	5408

The epidemiological investigation suggests that clusters 1, 2, and 3 may originate from an outbreak in one high school in Nagayo Town. Most of the positive cases had a history of traveling to other prefectures (Ehime, Fukuoka, Okinawa, Shizuoka, etc.). Subsequent infections within families or close-contact people may result in a long duration of all four clusters.

### Space-time clusters at machi-level in Nagasaki Prefecture and DIDs: Period 2 (4/1/2020–3/31/2021)

**Fig. 4:**
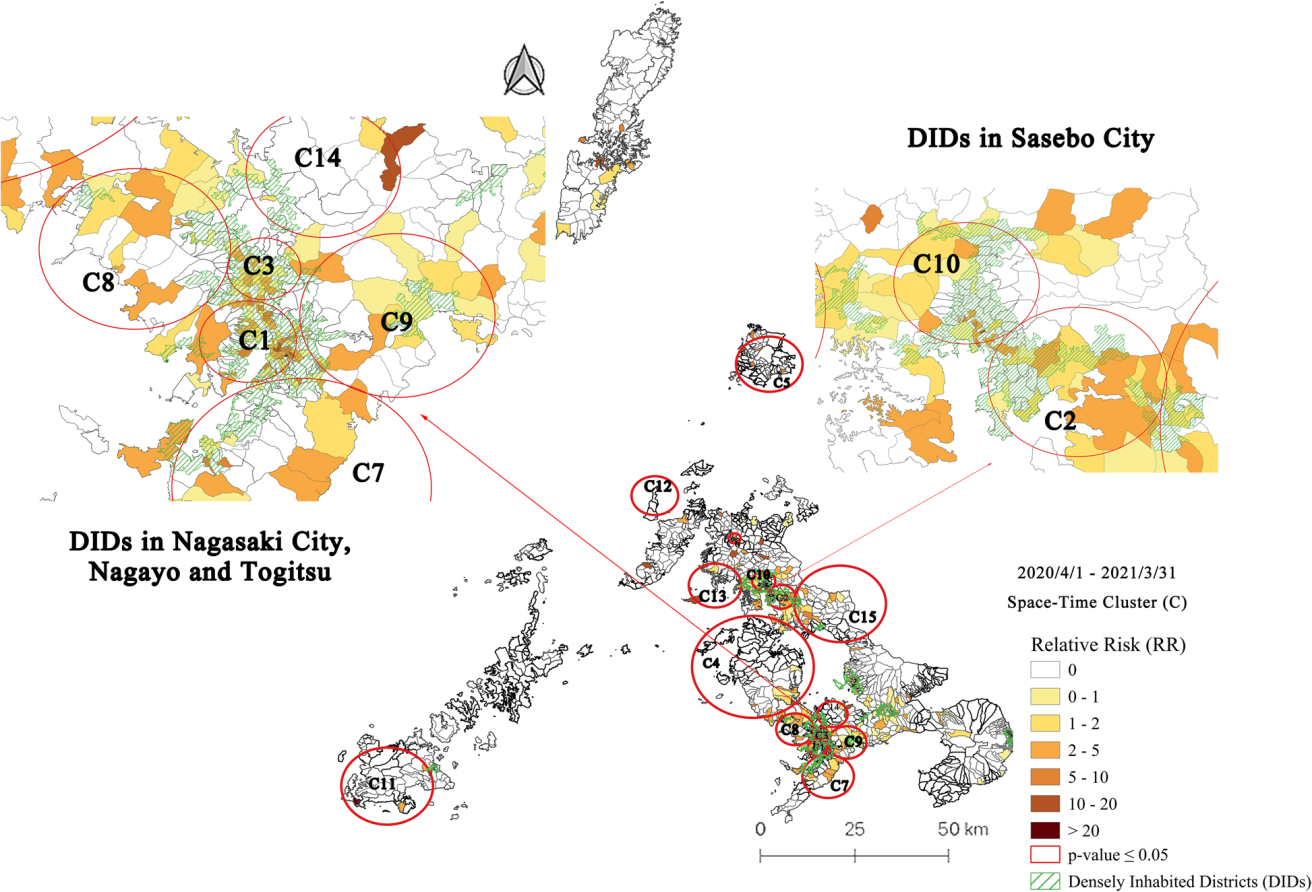
Emerging space-time clusters of COVID-19 cases at machi-level in Nagasaki Prefecture from 2020/4/1 to 2021/3/31

Period 2 includes the whole third wave of COVID-19 in Nagasaki Prefecture. Fifteen statistically significant emerging clusters were detected by March 31, 2021 (Fig. 4). Table 3 summarizes the characteristics of these clusters. The results showed a vigorous growth of COVID-19 infections across the mainland of Nagasaki Prefecture, mainly in Nagasaki City, Sasebo City, and Saikai City. The most likely cluster (C1) and cluster 3 were found in DIDs in Nagasaki City, consisting of many commercial districts and neighborhood units. Cluster 1 presented a relative risk (RR) of 9.22, with 216 observed cases compared to 26.37 as expected; 72 out of 107 machis in cluster 1 had a RR > 1. Around this cluster, there are five less-likely clusters (3, 7, 8, 9, 14). Cluster 2 was located in Sasebo City with a RR of 7.21; it involved 137 cases and 30 machis, of which 21 machis had a RR > 1. Other clusters were spread among Saikai City and Sasebo City, while three clusters were located away from the mainland on remote islands.

**Table 3:** Characteristics of emerging space-time clusters of COVID-19 cases at machi-level in Nagasaki Prefecture from 2020/4/1 to 2021/3/31

Cluster	Period	Duration (Days)	Radius (km)	Observed Cases	Expected Cases	RR	*P*-value	Machi (Total)	Machi (RR > 1)	Population
1	2020/12/9–2021/3/31	112	2.38	216	26.37	9.22	< 0.001	107	72	67,548
2	2021/1/3–2021/3/31	87	3.49	137	20.4	7.21	< 0.001	30	21	67,107
3	2020/12/21–2021/3/31	100	1.8	112	23.72	4.96	< 0.001	39	24	67,998
4	2020/12/13–2021/3/31	108	15.03	93	17.46	5.57	< 0.001	80	28	46,378
5	2020/12/28–2021/3/31	93	8.14	51	7.2	7.27	< 0.001	68	21	22,161
6	2020/12/20–2021/3/31	101	1.47	21	0.62	34.37	< 0.001	6	5	1755
7	2020/12/10–2021/3/31	111	6.36	92	25.6	3.74	< 0.001	43	20	66,175
8	2020/12/10–2021/3/31	111	4.68	86	25.42	3.51	< 0.001	31	14	65,704
9	2020/12/10–2021/3/31	111	4.78	76	25.37	3.09	< 0.001	35	16	65,587
10	2020/12/24–2021/3/31	97	2.84	65	19.61	3.41	< 0.001	75	27	57,924
11	2021/1/6–2021/3/31	84	11.36	31	4.64	6.78	< 0.001	38	8	15,812
12	2021/1/13–2021/3/31	77	5.72	18	1.21	15.06	< 0.001	4	3	4479
13	2020/12/19–2021/3/31	102	6.47	26	4.2	6.27	< 0.001	9	3	11,805
14	2020/12/10–2021/3/31	111	3.78	46	12.91	3.63	< 0.001	16	8	33,375
15	2020/12/28–2021/3/31	93	11.28	56	21.72	2.63	< 0.001	76	16	66,898

The majority of clusters lasted for the entirety of Period 2 (an average of 100 days). During Period 2, three consecutive holidays (new year, adult day, spring equinox day) had promoted traveling between Nagasaki Prefecture and other prefectures. From December 2020 to the beginning of 2021, cluster infections were found in public places, including restaurants, welfare services for people with disabilities, schools, welfare facilities for the elderly, nursery schools, stores, and administrative buildings. Epidemiological investigation showed that quite a few early cases in this period had an out-of-prefecture history of traveling. Most of the cases had close contact with other COVID-19 positive patients.

### Space-time clusters at machi-level in Nagasaki Prefecture and DIDs: Period 3 (4/1/2020–6/30/2021)

**Fig. 5:**
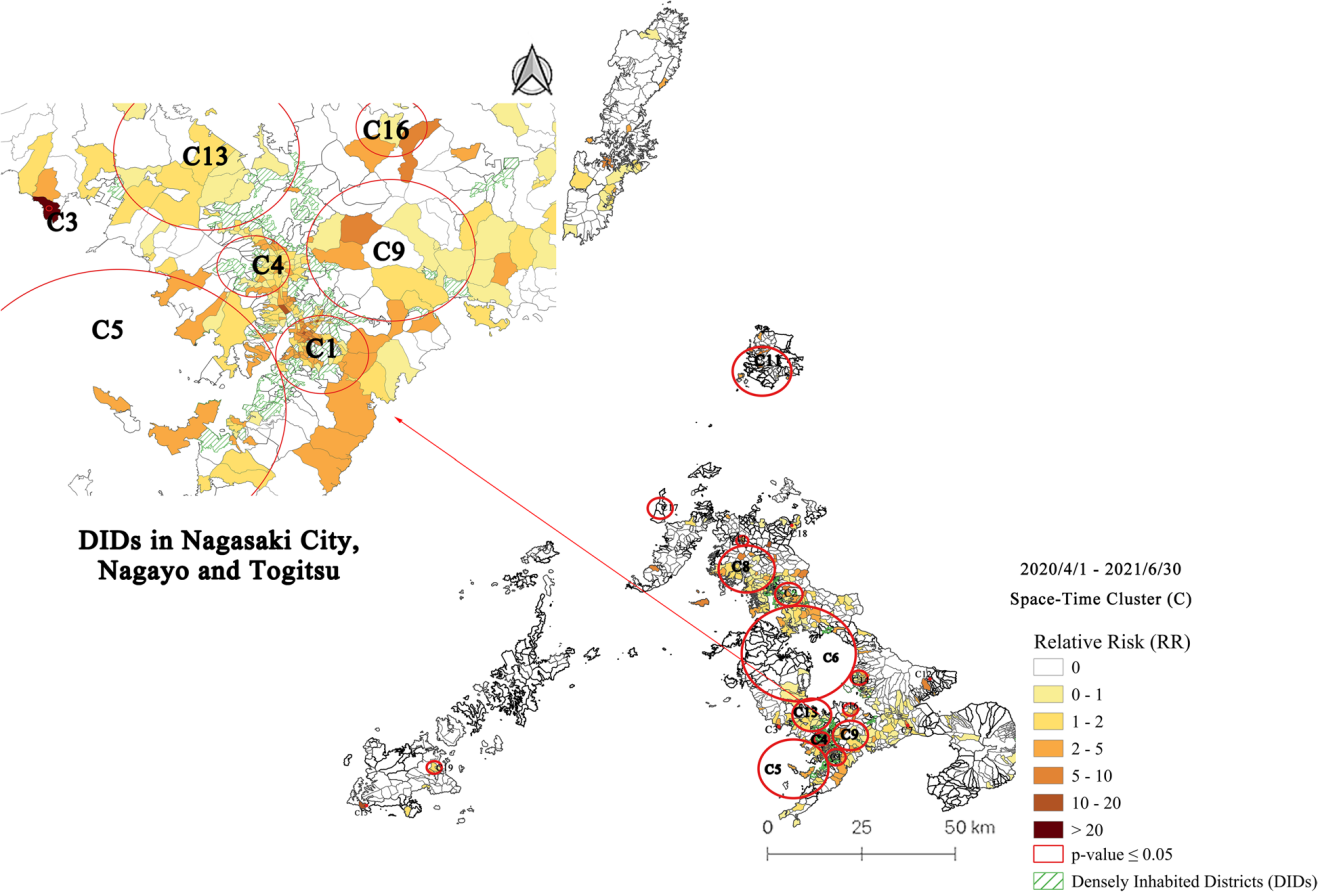
Emerging space-time clusters of COVID-19 cases at machi-level in Nagasaki Prefecture from 2020/4/1 to 2021/6/30

For the third study period, along with the fourth wave of COVID-19, 19 statistically significant clusters were detected by June 30, 2021, as shown in Fig. 5. Related cluster characteristics are shown in Table 4. Period 3 showed an expansion of spatial clusters to the northern part of Nagasaki Prefecture. Some clusters with small magnitudes or containing a single machi (cluster radius = 0) were found in Isahaya City (C7, C12), Omura City (C14), Matsuura City (C18), and Goto Island (C15, C19). Nagasaki City remains the most affected area in Nagasaki Prefecture, with five clusters identified with 909 observed cases and 152 machis (out of 214) with a RR > 1. The most likely cluster (C1) and cluster 4 in Nagasaki City have become more consolidated as the number of cases has increased since Period 2, which could explain cluster 13 in Nishisonogi District, cluster 2 in Sasebo City, cluster 11 in Iki Island, and cluster 17 in Hirado City. It suggests a continuation of outbreaks among local crowds. Besides the clusters mentioned above, starting from Period 2, new clusters appeared in Nagasaki City (C3, C5, C9) and Sasebo City (C8) around the DIDs of both cities.

**Table 4:** Characteristics of emerging space-time clusters of COVID-19 cases at machi-level in Nagasaki Prefecture from 2020/4/1 to 2021/6/30

Cluster	Period	Duration (Days)	Radius (km)	Observed Cases	Expected Cases	RR	*P-value*	Machi (Total)	Machi (RR > 1)	Population
1	2020/12/9–2021/6/30	203	2.39	433	73.83	6.6	< 0.001	98	82	68,358
2	2021/1/3–2021/6/30	178	3.37	260	63.75	4.34	< 0.001	37	28	67,268
3	2021/3/31–2021/6/30	91	0	36	0.34	106.89	< 0.001	1	1	699
4	2020/12/21–2021/6/30	191	1.88	237	67.96	3.68	< 0.001	40	27	66,876
5	2021/4/23–2021/6/30	68	8.61	113	24.36	4.77	< 0.001	46	28	66,672
6	2020/12/19–2021/6/30	193	14.01	187	68.46	2.84	< 0.001	89	37	66,654
7	2021/4/16–2021/6/30	75	0	23	0.4	57.78	< 0.001	1	1	996
8	2021/5/28–2021/6/30	33	23.79	71	12.17	5.94	< 0.001	89	28	67,622
9	2021/4/26–2021/6/30	65	4.34	90	23.79	3.86	< 0.001	29	14	68,094
10	2020/12/20–2021/6/30	192	1.47	28	1.79	15.74	< 0.001	6	5	1755
11	2020/12/28–2021/6/30	184	7.16	65	15.06	4.38	< 0.001	55	23	15,377
12	2021/4/14–2021/6/30	77	0	9	0.037	242.81	< 0.001	1	1	90
13	2020/12/10–2021/6/30	202	4.78	147	72.92	2.06	< 0.001	20	10	67,843
14	2021/4/19–2021/6/30	72	2.09	47	13.87	3.42	< 0.001	20	8	35,891
15	2021/1/12–2021/6/30	169	0	8	0.23	34.8	< 0.001	1	1	256
16	2021/1/14–2021/6/30	167	1.83	11	1.19	9.27	< 0.05	3	2	1338
17	2021/1/13–2021/6/30	168	3.06	19	4.01	4.76	< 0.05	4	3	4479
18	2021/4/26–2021/6/30	177	0	10	0.94	10.65	< 0.05	1	1	2694
19	2021/5/1–2021/6/30	176	1.85	16	3.13	5.13	< 0.05	7	4	9700

Several cluster infections were reported in welfare services for persons with disabilities, hospitals, restaurants, and self-defense force stations during mid-April. Soon after the “Golden Week” at the end of April, the Nagasaki government declared a state of emergency from May 12 to May 28, 2021 (level 5 alert) to stem the rapid increase of daily confirmed COVID-19 cases.

### Space-time clusters at machi-level in Nagasaki Prefecture and DIDs: Period 4 (2020/4/1–2021/9/8)

**Fig. 6:**
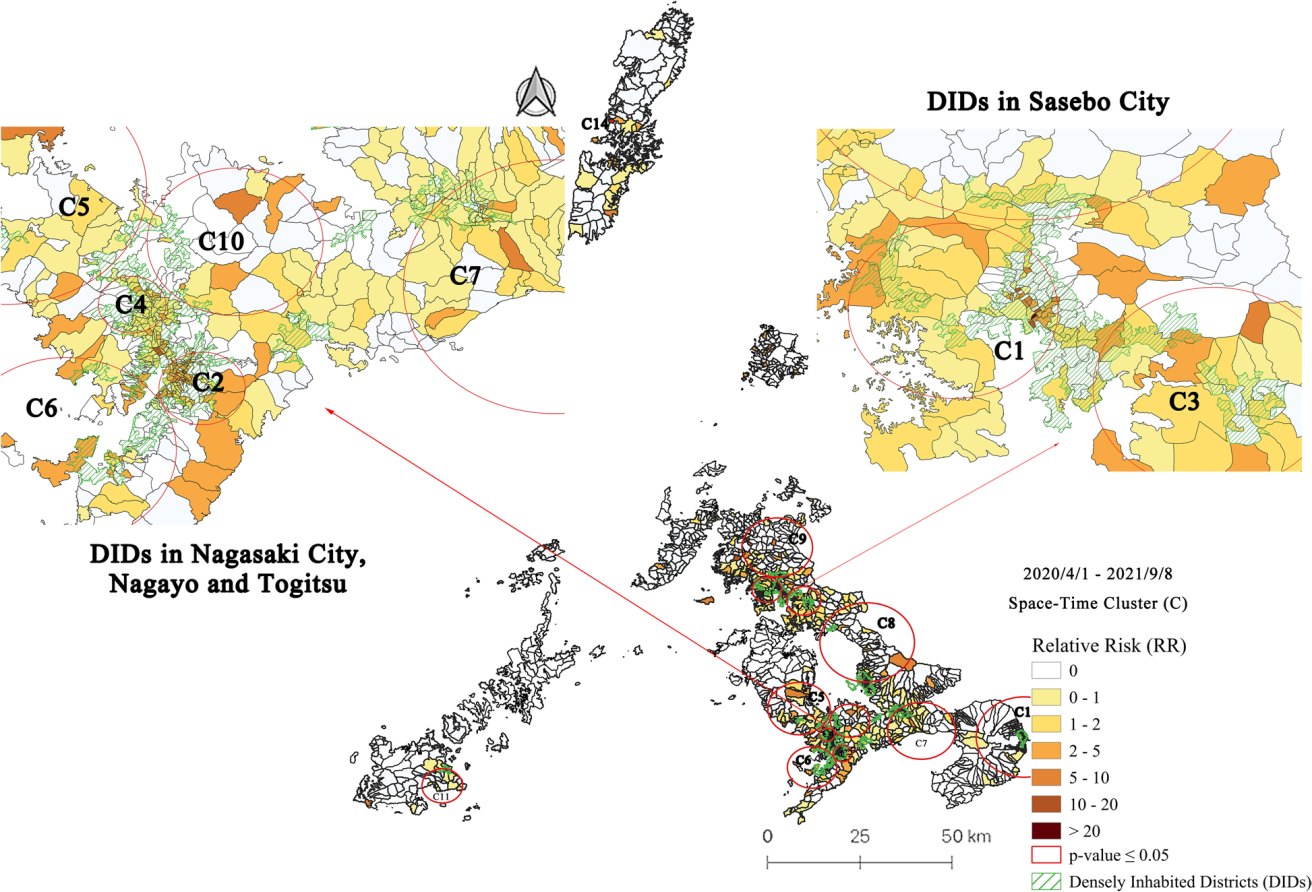
Emerging space-time clusters of COVID-19 cases at machi-level in Nagasaki Prefecture from 2020/4/1 to 2021/9/8

Up to September 8, 2021, including the fifth wave of COVID-19 infection, the prospective space-time scan statistic identified 13 statistically significant clusters (Fig. 6). The characteristics of these 13 clusters are summarized in Table 5. The most likely cluster (C1) in Period 4 is in the DIDs of Sasebo City, with 268 observed cases compared to 22.31 expected, a relative risk of 12.58, and 49 out of 77 machis had a RR > 1. It is worth noting that some “ancient” clusters persist in the same regions in the DIDs of Nagasaki City (C2, C4) and Sasebo City (C3) since Period 2, and cluster 6 (Nagasaki City) since Period 3. Until early September, cluster distribution became more homogeneous throughout the prefecture. It has to extend over almost everywhere on the mainland of Nagasaki Prefecture, except for Hirado City and Saikai City. Several new clusters have arisen in the latest wave of COVID-19; clusters 5 and 10 in the northern part of Nagasaki City and Nishisonogi District, clusters 9 in Sasebo City, cluster 7 in Isahaya City, cluster 8 in Higashisonogi Town/Omura City, cluster 11 in Goto Island, and cluster 14 in Tsushima Island.

**Table 5:** Characteristics of emerging space-time clusters of COVID-19 cases at machi-level in Nagasaki Prefecture from 2020/4/1 to 2021/9/8

Cluster	Period	Duration (Days)	Radius (km)	Observed Cases	Expected Cases	RR	*P-value*	Machi (Total)	Machi (RR > 1)	Population
1	2021/7/26–2021/9/8	44	3.65	268	22.31	12.58	< 0.001	77	49	65,595
2	2020/12/21–2021/9/8	261	2.39	556	133.16	4.54	< 0.001	96	74	67,231
3	2021/1/3–2021/9/8	248	4.21	405	125.19	3.41	< 0.001	31	19	66,506
4	2021/1/4–2021/9/8	247	1.87	252	69.81	3.74	< 0.001	39	28	66,433
5	2021/8/4–2021/9/8	35	7.69	130	18.34	7.24	< 0.001	42	16	67,402
6	2021/4/23–2021/9/8	138	6.11	232	68.43	3.5	< 0.001	45	24	65,127
7	2021/8/1–2021/9/8	38	8.36	111	19.99	5.65	< 0.001	69	21	67,786
8	2021/7/26–2021/9/8	44	11.65	115	23.24	5.03	< 0.001	87	21	68,308
9	2021/8/2–2021/9/8	37	8.67	98	19.54	5.09	< 0.001	87	25	68,027
10	2021/8/3–2021/9/8	36	4.86	86	18.6	4.68	< 0.001	31	12	66,504
11	2021/8/16–2021/9/8	23	5.03	29	2.38	12.22	< 0.001	22	3	13,143
13	2021/8/6–2021/9/8	33	11.81	54	17.33	3.14	< 0.001	134	9	67,441
14	2021/8/16–2021/9/8	23	0	3	0.0089	337.64	< 0.05	1	1	49

During this period, cluster infections frequently occurred in public places (restaurants, schools, nursery schools, city halls, driving schools, etc.). A provincial-level state of emergency was declared from August 19 until September 12, 2021. In the interim, the Nagasaki Governor decided to implement extra priority measures to prevent the spread of disease in Nagasaki City and Sasebo City from August 27, 2021.

### Space-time clusters at machi-level in Nagasaki Prefecture and DIDs: Period 5 (2020/4/1–2022/4/7)

**Fig. 7:**
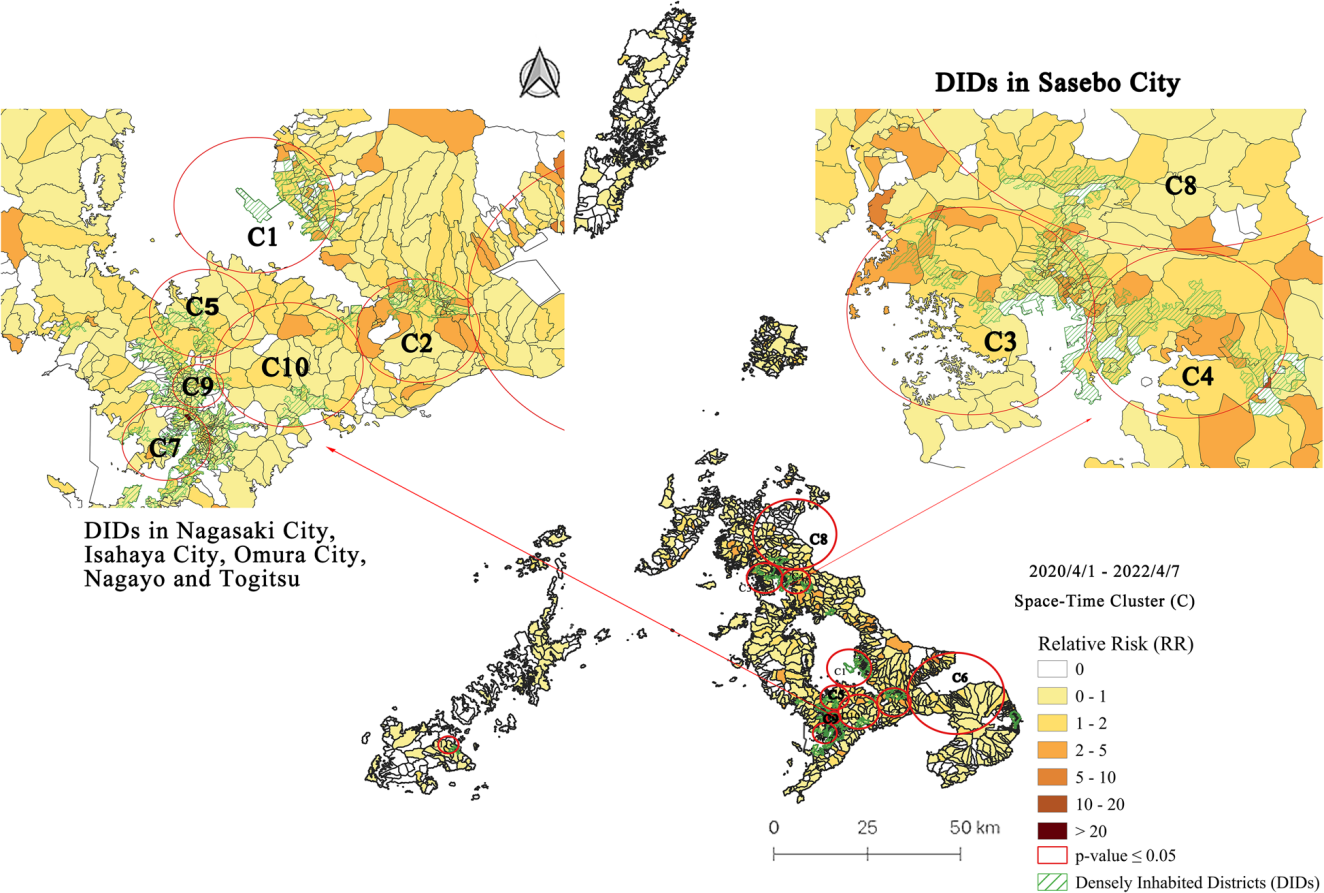
Emerging space-time clusters of COVID-19 cases at machi-level in Nagasaki Prefecture from 2020/4/1 to 2022/4/7

Our study period ends on April 7, 2022. During this period, including the sixth wave of the COVID-19 infection, the prospective space-time scan statistics identified 11 statistically significant clusters (Fig. 7), and summarized in Table 6. During this period, the Omicron variant of COVID-19 caused the tallest spikes in COVID-19 infections in Nagasaki Prefecture. The most likely cluster (C1) in Period 6 was in Omura City, with 2038 observed cases compared to 199.4 expected, a relative risk of 10.9, and 37 out of 61 machi had a RR > 1. The space-time clusters were largely concentrated in the DIDs of Nagasaki City (C5, C7, C9, C10), Isahaya City (C2), Omura City (C1), Sasebo City (C3, C4), and Goto Island (C11). Additionally, two other secondary clusters were located in Unzen City (C6) and Matsuura City (C8). All the clusters during this period were from the mid-January to April, 2022.

**Table 6:** Characteristics of emerging space-time clusters of COVID-19 cases at machi-level in Nagasaki Prefecture from 2020/4/1 to 2022/4/7

Cluster	Period	Duration (Days)	Radius (km)	Observed Cases	Expected Cases	RR	*P-value*	Machi (Total)	Machi (RR > 1)	Population
1	2022/1/17–2022/4/7	80	5.61	2038	199.4	10.9	< 0.001	61	37	68,337
2	2022/1/12–2022/4/7	85	4.28	2059	208.01	10.56	< 0.001	55	39	67,146
3	2022/1/11–2022/4/7	86	4.51	1941	213.67	9.65	< 0.001	87	68	68,182
4	2022/1/12–2022/4/7	85	3.66	1888	203.15	9.85	< 0.001	33	26	65,577
5	2022/1/13–2022/4/7	84	3.64	1424	196.08	7.24	< 0.001	18	13	64,040
6	2022/1/12–2022/4/7	85	12.26	1233	209.81	6.09	< 0.001	95	41	67,726
7	2022/1/12–2022/4/7	85	3.06	1236	212.11	6.04	< 0.001	114	48	68,470
8	2022/1/12–2022/4/7	85	10.56	1152	210.16	5.66	< 0.001	112	32	67,839
9	2022/1/12–2022/4/7	85	1.77	1134	208.99	5.6	< 0.001	45	17	67,464
10	2022/1/12–2022/4/7	85	5.15	951	186.11	5.25	< 0.001	32	17	60,076
11	2022/1/18–2022/4/7	79	2.58	58	21.52	2.7	< 0.05	17	3	12,180

The first case of the Omicron variant was confirmed on January 3, 2022 in Nagasaki Prefecture (Fig. 1c), and was followed by a sharp surge of the infection that quickly spread across the prefecture at a speed three times higher than that of the previous wave. The epidemiological investigation showed that approximately 80% of the first cases were from outside the prefecture, and most of the secondary infections were related to social interactions of people. Frequent cluster infections were reported in welfare facilities, hospitals, restaurants, schools, and other public places since January 7, 2022. To prevent the situation from deteriorating, a province-level state of emergency with extra priority measures was declared from January 21 to March 4, 2022 in Nagasaki Prefecture.

## Discussion

This study applied prospective space-time scan statistics to identify active or emerging clusters of COVID-19 in Nagasaki Prefecture at the machi level, presenting results for five consecutive periods. In each period, significant wave/waves of COVID-19 infection were covered, which permitted us to glimpse the COVID-19 infection’s progression in Nagasaki Prefecture. As far as we are sure, this is the first study that utilizes space-time scan statistics to detect COVID-19’s emerging clusters and get a full image of the COVID-19 situation since the epidemic’s beginning in Nagasaki Prefecture.

Prospective space-time scan statistics are a valuable surveillance tool to monitor disease outbreaks [26]. It conducts rapid statistical analysis to understand COVID-19’s highest risk areas and how the risk levels will change during this pandemic [27]. Several studies used this method in the COVID-19 surveillance system [16, 18, 19, 27, 28]. The prospective approach used in this study has been demonstrated to be useful for rapid monitoring of COVID-19’s space-time patterns in Nagasaki Prefecture. Thus, this would enable rapid analysis and a more dependable understanding of high-risk regions, contributing to the establishment of an effective vaccination plan and NPIs within Nagasaki Prefecture.

Before December 2020, only four clusters emerged, located dispersedly on Nagasaki Prefecture’s mainland and one on the distant island of Tsushima. As reported by the retrospective contact tracing investigations, these clusters represented multiple early cluster infections in medical centers and high schools, and most of the positive cases were traceable and the closed contacts were under segregated. At this time, the NPIs that were taken concentrated on keeping social distance, self-restriction, and avoiding the “3 C’s” [29]. Likewise, the first national-wide state of emergency (April 7 to May 25, 2020) had significantly limited growth in daily confirmed COVID-19 cases.

In comparison to the first wave of COVID-19, which occurred from March to April, the number of people infected with an unidentified transmission route infection sharply increased in the ensuing months. To resume the economic consequences caused by the first nationwide state of emergency, no other state of emergency was issued during the second wave in late June 2020 [30]. Alternatively, certain standards were formulated by the Nagasaki Governor for responding to the COVID-19 pandemic to guarantee an adequate medical care provision system in Nagasaki Prefecture (Supplementary material 1).

The third wave (Period 2) of COVID-19 in Nagasaki Prefecture started in early December 2020, roughly 1 month later than in prefectures with big cities like Tokyo, Osaka, and Fukuoka [30], even though the local and national governments requested residents to avoid unnecessary travel between prefectures, especially those from prefectures with high infection risk. Mild restrictions policies, however, could not restrain the disease transmission because interregional mobility speeded up infection’s geographical expansion from urban prefectures with numerous infectious people to rural prefectures [31]. A “Go-To” campaign to encourage travel and dining out started on July 22, 2020, and a consecutive New Year’s Day (Fig. 1b) may have prompted an infected/ suspicious individual influx from neighboring prefectures into Nagasaki Prefecture. The contact tracing investigation showed that some of the early confirmed cases in early December had a history of traveling to other prefectures, and domestic travel to the epicenter of the epidemic at the time was a risk factor for the spread of COVID-19 infection [13]. In December, several cluster outbreaks in restaurants, stores, welfare facilities for the elderly, and clinics were reported. Karako et al. [30] discussed that during the third wave of COVID-19, people did not reduce their activity level even if the infected patients grew rapidly. As a prefecture with plenty of rural regions/remote islands with scarce healthcare resources, like Nagasaki Prefecture [32], infections in different locations during the daytime could spread quickly to their residential communities. It was not until January 16 that the Nagasaki government elevated the infection stage to level 4 (Nagasaki City to level 5), following the guidelines [9] depending on bed usage for patients with COVID-19 (Fig. 1b). In stage 4/5, demands for shortening business hours, refraining from unnecessary and unurgent travel to other prefectures, and refraining from holding events were applied according to the infection situation in different municipalities. After the comparatively fierce countermeasures, there were no more reported cluster outbreaks, and the number of daily infected went down to a low level.

During the fourth wave (Period 3), half of the clusters identified by the space-time scan statistics originated from the third wave (Period 2). The most likely clusters in the two study periods were located in the same DID regions of Nagasaki City; more specifically, the overlapping parts of the two most likely clusters were the busiest commercial areas of Nagasaki City. The most likely cluster of Period 3 also started on December 9, 2020, the same time as in Period 2. The secondary clusters of the two study periods, which were located in Nagasaki City, Sasebo City, Saikai City, Iki Island, and Ikitsuki Island (Hirado City), showed high overlapping both in terms of geography and timing. Other “young clusters” located in Isahaya City, Omura City, and Matsuura City, suggested an infection expansion across most districts in Nagasaki Prefecture. In Nagasaki Prefecture, the vaccination program against COVID-19 for health workers and older people started in early April of 2021, and the citizens were expected to change their behaviors [33], mainly reflected in reducing in population density [13]. However, the Nagasaki Government did not stopped the “Heart Breathing” campaign to promote tourism within Nagasaki Prefecture until April 19 (Fig. 1b). The results from Periods 2 and 3 indicated that restricting interregional mobility without limiting intraregional mobility would slow down, but not stop, the infection spread to rural areas. Although the state of emergency declaration may have reduced the number of infections in the workplace, approximately 2 weeks after the declaration, an increase in domestic infections and the unknown route of community-acquired infections’ transmission rose [34].

During the fifth wave of COVID-19 (Period 4), 13 emerging clusters were identified at the machi level in Nagasaki Prefecture (Fig. 6). In Nagasaki Prefecture’s mainland, DIDs in Nagasaki City and Sasebo City were experiencing a moderate to high level of risk. However, less populated areas, such as villages, mountains, and remote islands were at low to no risk of COVID-19 infection. It is worth noting that the emergence of Cluster 2–4 in early December 2020 was an indication that the DIDs were the high-risk areas once the infection diffused in the region due to COVID-19’s transmission dynamics [35]. The distinction in the fifth wave was that more clusters in non-DID areas with short durations emerged and were less centralized. The possible reasons for this are the resuming of the “The Heart Breathing” trip campaign, a relatively low rate of fully vaccinated people (Fig. 1a) and vaccination intention [36, 37], and the confirmation of the COVID-19 Delta variant [38] on July 21. In the first and second waves, retrospective contact-tracing investigations revealed the source patients and clusters of more than 60% of confirmed cases. After a certain period, especially with the rapid growth of daily cases, the outbreaks in hospitals, healthcare facilities, and schools, posed significant challenges to large-scale case investigation and contract tracing [39] with inadequate human resources. The number of reported cases without an identified epidemiological link and unrecognized community transmission chains increased [40].

The first case of the Omicron variant was confirmed on January 4, 2022 in Nagasaki Prefecture, and the recorded infections were dominated by the variant within the prefecture in the following 2 weeks. Subsequently, a more contagious Omicron sublineage BA.2 [41] was confirmed on February 28, 2022. This highly infectious Omicron variant showed a tendency to trigger cluster infections in a crowd. Indeed, during the sixth wave of COVID-19, 9 out of 11 emerging clusters were in the DIDs areas of Nagasaki Prefecture. Factors such as less severe presentation in people infected with the Omicron variant [42], a percentage of around 80% of fully vaccinated population against COVID-19 since the end of September 2021 in the region, were supposed to release the pressure on the healthcare system. However, the shortage of hospital beds and medical staff forced the Nagasaki government to issue a province-level state of emergency with extra priority measures on January 21, 2022 (Fig. 1c). The daily rapid growing of COVID-19 cases, waning COVID-19 vaccine effectiveness [43], and the increase in the number of infected children during the Omicron wave could have diminished the effects of interventions implemented in the previous COVID-19 waves [44].

Nagasaki Prefecture ranks at the top among prefectures with the highest aging population in Japan, according to official Japanese government projections, and older adults with underlying conditions are the most vulnerable groups to COVID-19 [45, 46]. Furthermore, the insufficient medical resources in the remote islands and rural areas exposed Nagasaki Prefecture to the COVID-19 surge. Moreover, the allocation and efficient COVID-19 booster vaccination program in these areas could be a difficult point. The economic cost of restrictions on business activities, tourism, and work was not very prohibitive. Therefore, developing policies aimed at balancing epidemiological performance and economic damage is important [47]. Several studies revealed that the Japanese spontaneously changed their behavior [33, 48–50], but the number of countryside trips did not decrease [13]. Even without mandatory punishment for travelling across prefectures, the declaration produced a lockdown-like effect in rural prefectures in the sense that the risk level in high-risk areas rose, and that in low-risk areas dipped after the declaration [14]. Furthermore, the most significant implication of restricting interregional mobility was avoiding an epidemic peak overwhelming the existing healthcare services. Meanwhile, coordinated and targeted NPIs could help to reduce transmission to the local community [51].

In this study, the emerging clusters analysis of COVID-19 could capture the local cluster outbreaks in time and identify the high-risk areas (Supplementary material 2), making this method a worthwhile surveillance measure for monitoring disease progression. Therefore, we suggest that prospective scanning statistics be applied in the suite of tools available to policymakers and public health departments. It would be helpful for the allocation of adequate health resources, vaccination programs, early planning NPIs measures, intensive contact tracing, and inter−/intra-regional restrictions at appropriate time.

Despite its strengths, our study has several limitations. First, the machi-level address was used rather than the residential address for the positive cases in this study. It is premised on the population’s homogenous distribution. However, the mountainous and multi-island terrain contribute to the heterogeneity and complexity of the Nagasaki Prefecture population. Thus, the minimum radius setting could lead to a cluster with a large radius when sporadic cases are reported from rural areas. Therefore, additional contact tracing for transmission chains is needed for the cases in clusters in these regions. Second, our analysis did not adjust for age, despite COVID-19 being more severe for the elderly and those with chronic diseases [52]. Although experience worldwide has shown that COVID-19 can affect all age groups [27], future research should include younger age groups to examine the efficiency of NPI implementation. Third, we used the confirmed number of COVID-19 cases instead of positivity rate in the spatial-temporal cluster analysis due to the unavailability of data. Thus, some clusters detected in the early months could simply be a function of testing effort. Future studies should use positivity rate, as well as more variables to demonstrate transmission dynamics. Finally, the SaTScan’s application for space-time scan statistics. This approach uses cylindrical shapes to represent the cluster; they, however, are probably not the COVID-19 clusters’ shapes; more study on developing irregular search windows for disease detection is encouraged.

## Conclusions

This study gives an overall analysis of the transmission dynamics of the COVID-19 pandemic based on the number of machi-level daily cases in Nagasaki Prefecture. Emerging space-time clusters have been identified in six consecutive waves in the COVID-19 pandemic from April 1, 2020 to April 7, 2022. We found that DIDs in Nagasaki City and Sasebo City continued to be the highest-risk areas, with the highest number of emerging clusters since December 2020.

Space-time scan statistics support disease surveillance with reliable and timely information. Once the emerging outbreaks lead to an infection expansion, it could become a warning of community transmission. Besides, the findings in different waves can serve as references for subsequent pandemic prevention and control. This approach helps the health authorities track and investigate outbreaks of COVID-19 that are specific to these environments, particularly in rural areas where healthcare resources are scarce.

## Supplementary Information

**Additional file 1.**

**Additional file 2.**

## Data Availability

The data used in this study were provided by the information center for infectious diseases of the Nagasaki Prefectural Institute of Environment and Public Health. The datasets used and/or analyzed during the current study are available from the corresponding author on reasonable request.

## Abbreviations

COVID-19: Coronavirus disease 2019
DIDs: Densely inhabited districts
WHO: World Health Organization
NPIs: Non-pharmaceutical interventions
RR: Relative risk
