# Supplementary figures and images for: Exploring spatiotemporal patterns of COVID-19 infection in Nagasaki Prefecture in Japan using prospective space-time scan statistics from April 2020 to April 2022
Source: Arch Public Health. 2022 Jul 26;80:176. doi: 10.1186/s13690-022-00921-3 (PMC9315091; doi:10.1186/s13690-022-00921-3)

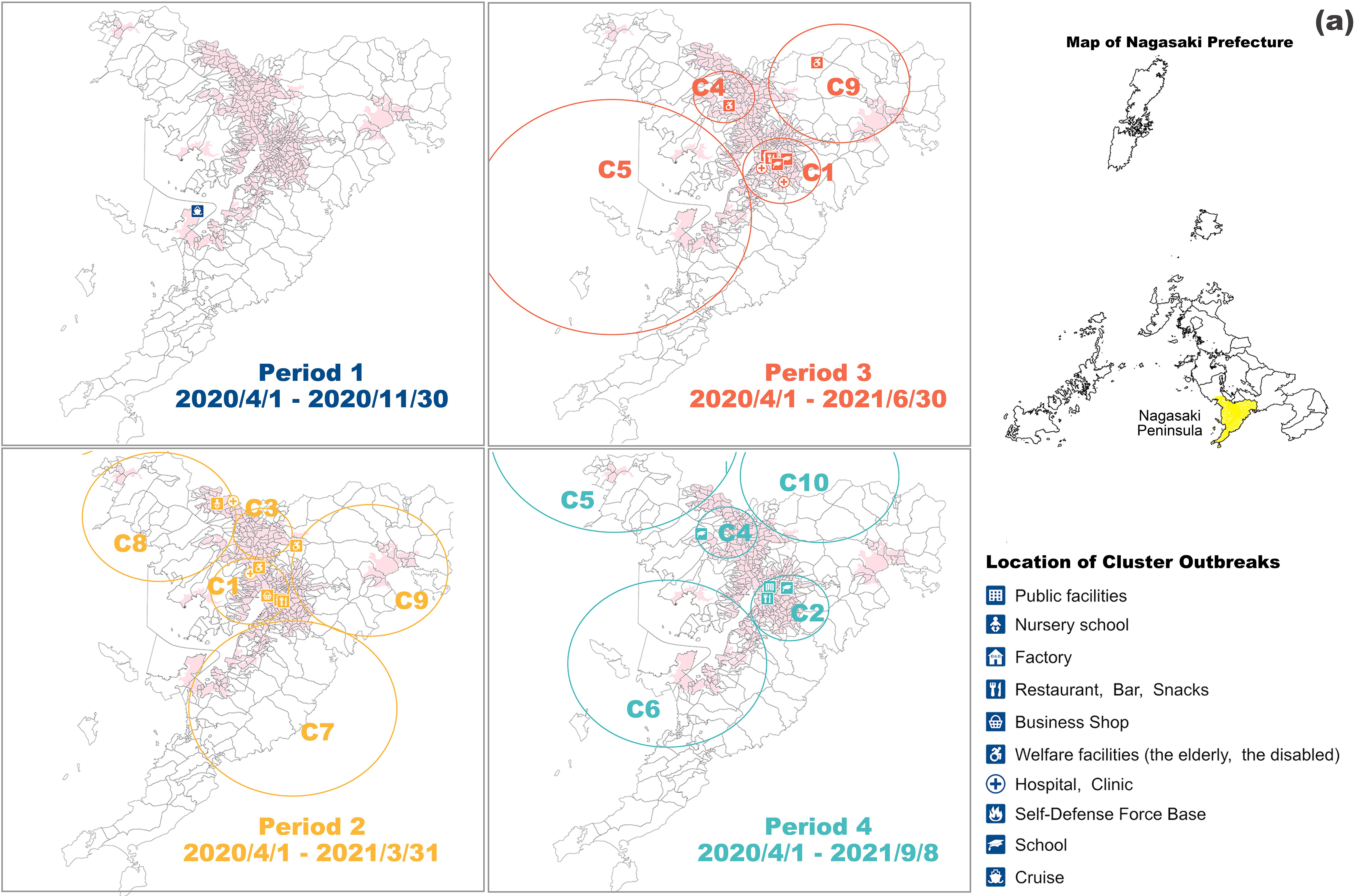

Supplement: Supplementary file 2 — Additional file 2. [file 13690_2022_921_MOESM2_ESM.zip › Supplementary material 2 (a).png]

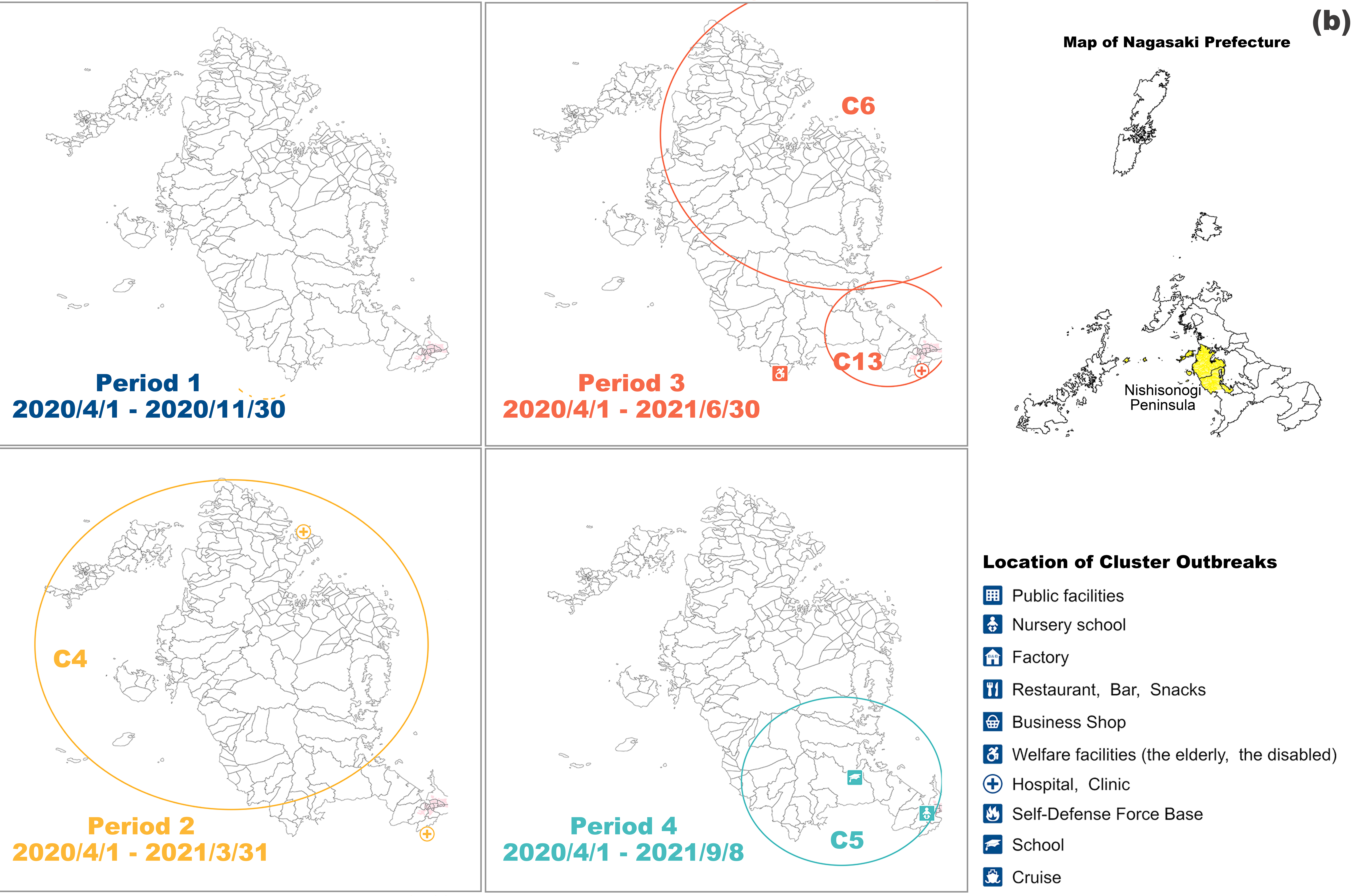

Supplement: Supplementary file 2 — Additional file 2. [file 13690_2022_921_MOESM2_ESM.zip › Supplementary material 2 (b).png]

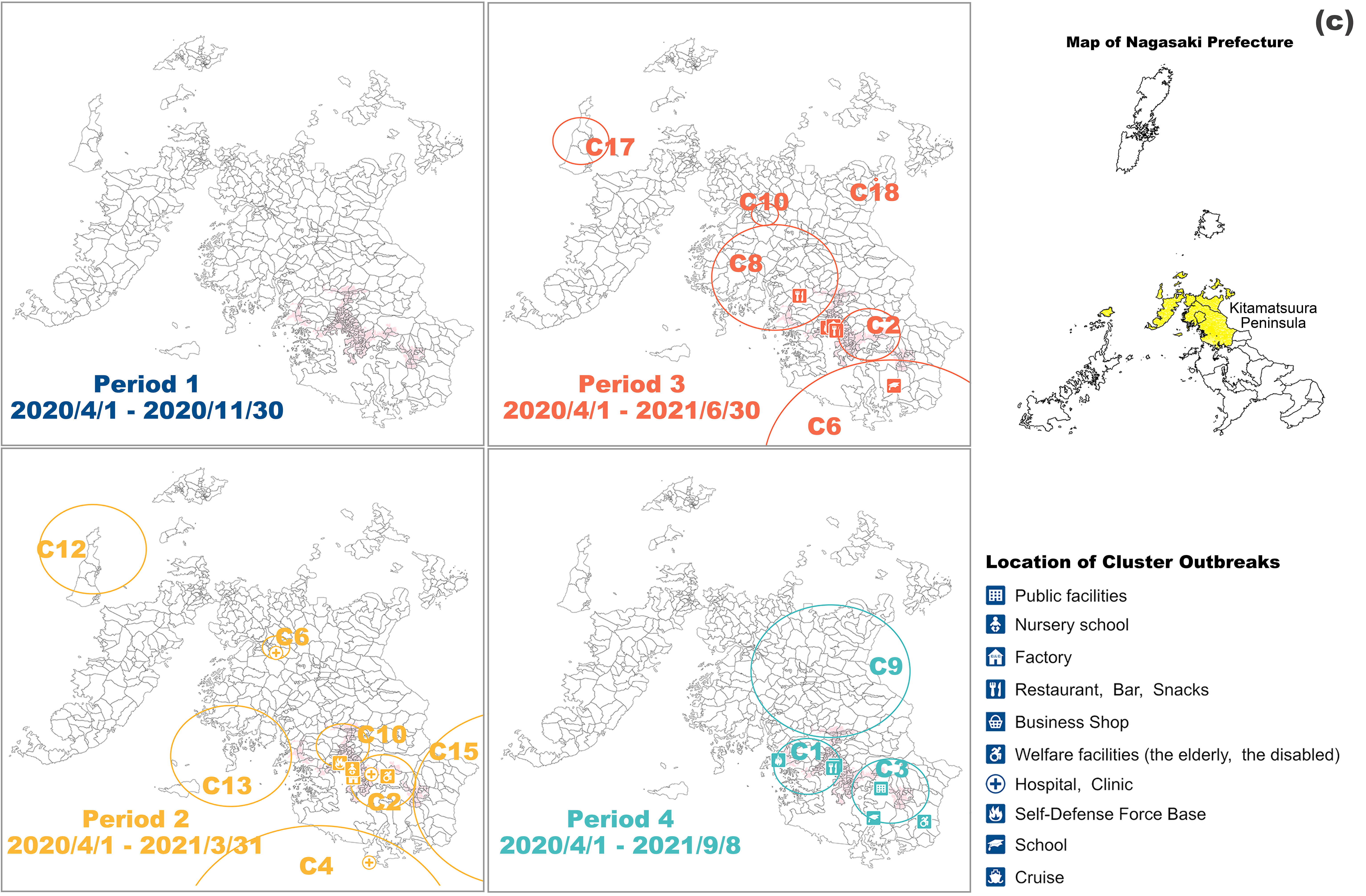

Supplement: Supplementary file 2 — Additional file 2. [file 13690_2022_921_MOESM2_ESM.zip › Supplementary material 2 (c).png]

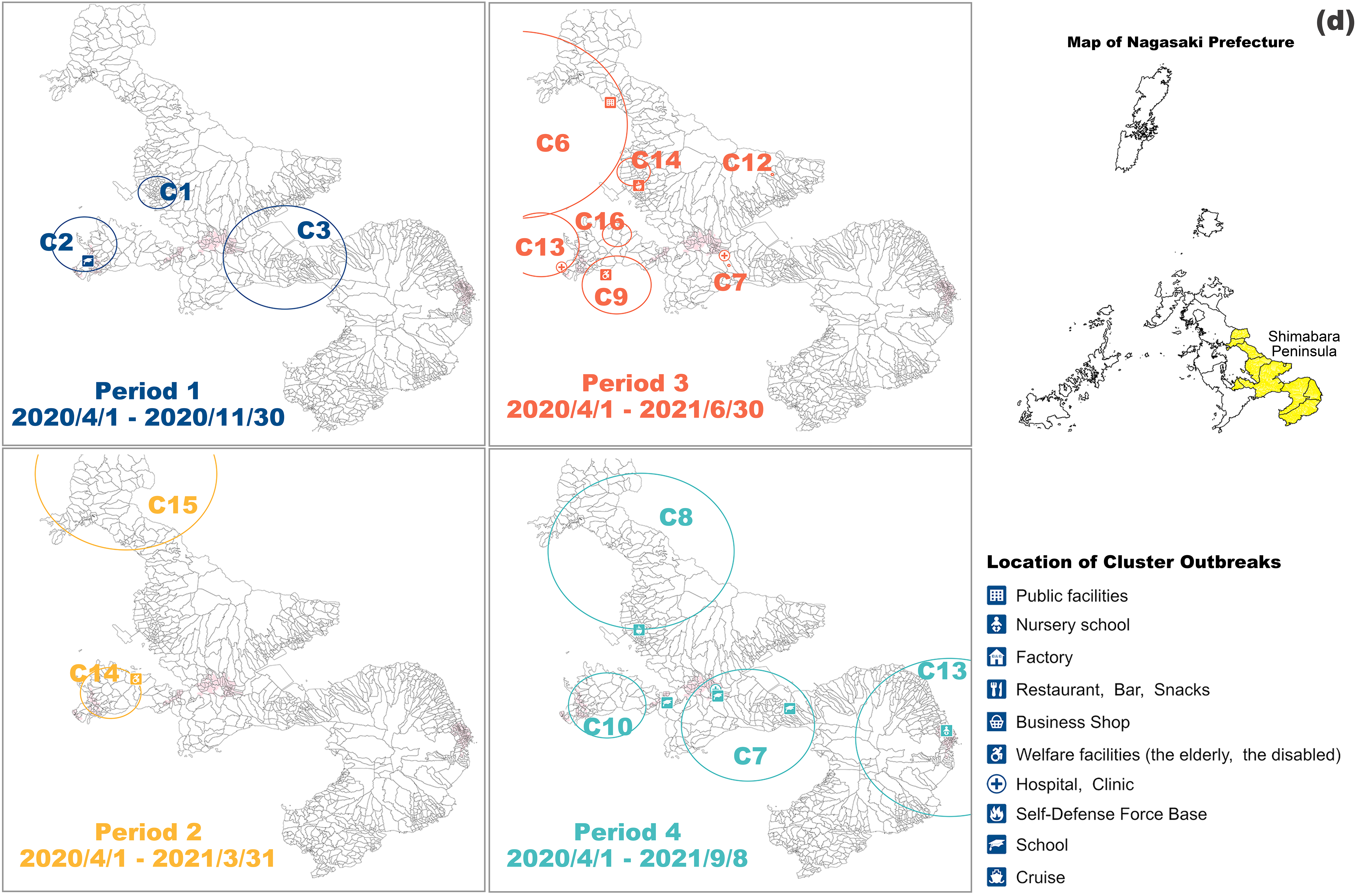

Supplement: Supplementary file 2 — Additional file 2. [file 13690_2022_921_MOESM2_ESM.zip › Supplementary material 2 (d).png]
